# Immediate postpartum use of long-acting reversible contraceptives in low- and middle-income countries

**DOI:** 10.1186/s40748-017-0063-z

**Published:** 2017-12-22

**Authors:** Margo S. Harrison, Robert L. Goldenberg

**Affiliations:** 1 Columbia University Medical Center, New York, NY USA; 2 622 W 168th St, PH 16-29, New York, NY 10032 USA

**Keywords:** Contraception, Long-acting reversible contraception, Immediate postpartum contraception

## Abstract

Globally, data show that many women of reproductive age desire to use modern family planning methods. Many of these women do not have access to modern contraceptives, which is termed their ‘unmet need’ for contraception. In low- and middle-income countries where total fertility rates can be high and many women have undesired fertility, or wish to increase their inter-pregnancy intervals, access to modern contraceptives is often inadequate.

The puerperium is a unique time for interventions to offer modern contraceptive methods. Having just given birth, women may desire contraceptives to prevent short-interval pregnancy, or further pregnancy, altogether. In high-, middle-, and low-income countries there has been an increased interest in the placement of long-acting reversible contraceptives at or immediately after delivery, regardless of delivery mode. These methods can provide women with highly effective contraception for years, can be manufactured at low cost, are generally well tolerated with a good safety profile, and do not require the user to remember to take them. Oral contraceptives and injectable medications require the patient to present to the clinic during a specific timeframe for follow-up care or a refill, and the clinic may not be proximate, affordable, or have the desired contraceptive in stock.

This document will review the currently published literature on the use of immediate postpartum long-acting reversible contraceptives (placed within two days of delivery) in low- and middle-income countries to report on the prevalence of use and satisfaction rates, and note the lack of data on cost and economic implications. We will also explore data on how future maternal, neonatal, and infant outcomes may be influenced by increased peripartum long-term contraceptive use.

## Background

Long-acting reversible contraception (LARC) includes copper and progesterone-laden intrauterine devices (IUDs) and progesterone-only contraceptive implants, per the World Health Organization (WHO) [1]. LARC is the most effective method of modern contraception and offers the advantages of a rapid return of fertility with removal and are user-independent. Once the devices are placed, the woman does not need to perform any action to support ongoing effective use of the contraceptive [2]. It should be noted that some sources, such as the National Institute for Healthcare Excellence (NICE) guidelines from the United Kingdom, include progesterone-only injectable contraceptives in the category of LARC; for the purposes of this review on the use of postpartum LARC in low- and middle-income countries (LMIC), the definition will be restricted to IUDs and implants per the WHO [3].

### Intrauterine devices

LARC includes two types of IUDs, the copper and levonorgestrel (LNG) IUDs. The copper IUD is comprised of polyethylene wrapped copper and has been approved by the Food and Drug Administration (FDA) in the United States of America (USA) for use up to ten years. The failure rate over that time period is 1.9 in 100 women [2]. The IUD reduces ovum and sperm transport and viability [2]. The device has minimal side effects, although heavy menstrual bleeding and dysmenorrhea have been cited [2].

The LNG IUD system is approved by the FDA for five years of consecutive use, has a one-year failure rate of 0.2 per 100 women, and prevents pregnancy by altering the function and consistency of cervical mucous, endometrial receptivity, and preventing ovulation in 58–63% of women [2]. The device elutes 20 μg of drug daily and has minimal systemic impact, although headaches, nausea, breast tenderness, depression, and ovarian cyst formation are reported side effects [2]. Additionally, patients have reported discontinuation due to oligo- or amenorrhea, spotting, and irregular bleeding patterns [2].

### Contraceptive implant

The contraceptive implant is also a progesterone-only method that elutes etonogestrel over a three-year period and interferes with proper functioning of the hypothalamic-pituitary-ovarian axis while also altering the endometrial lining and cervical mucous [2]. Reported side effects include weight gain, vaginitis, breast pain, acne, headaches, gastrointestinal disturbances, and complications related to implant removal and insertion [2]. Some patients also complain of unpredictable bleeding patterns, which accounts for an 11.3% rate of discontinuation; the typical-use pregnancy rate is reported as 0.05%, which is the effectiveness based on correct and consistent use [2].

### Epidemiology

United Nations (UN) data indicate that LARC methods are popular, globally [4]. Sterilization, IUDs, and implants are reported to account for 56% of contraceptive prevalence in 2015, and according to the same UN report, are accounting for a greater share of all contraceptive use with increased prevalence of modern methods around the world [3]. Use of different methods varies by region, however, with the highest use of implants and IUDs in Asia and North America [4].

### Postpartum use

Immediate postpartum LARC is defined, per the American College of Obstetrics and Gynecologists (ACOG), as placement of LARC prior to hospital discharge [5]. As ACOG is the professional organization representing obstetrician/gynecologists in the USA, its definition is applicable to a high-income setting where the majority of deliveries occur in facilities. For the purposes of this review, immediate postpartum LARC will be defined as placement of LARC within two days of delivery, regardless of setting or method of delivery, as the IUD can be placed up to 48 h postpartum and the implant can be placed any time if the woman is not breastfeeding; breastfeeding is not an absolute contraindication as discussed below [6].

### Safety

The US Medical Eligibility Criteria for Contraceptive Use, which was developed by the Centers for Disease Control and Prevention (CDC) and the WHO, classifies the safety of contraceptives under various circumstances [7]. Contraceptives are classified as category I through IV. I represents no restrictions to use, II represents a situation in which benefits outweigh risks, III represents a situation in which risks outweigh benefits, and IV represents an unacceptable health risk to the patient of using a given contraceptive in that medical circumstance [7]. Immediate postpartum use (defined by the CDC as placement within ten minutes of placental separation) of the copper IUD is designated as a category I. The LNG IUD is classified as category II because of theoretical concerns regarding the effect of progesterone on breastfeeding, although published data has not supported this concern [2, 7]. IUDs are contraindicated during this time period in women with chorioamnionitis, endomyometritis, or puerperal sepsis [2].

In non-breastfeeding women, the contraceptive implant is category I any time after childbirth, but again, similar to the LNG IUD, is category II for breastfeeding women due to concerns about breastmilk production if placed at less than four weeks postpartum [2]. Again, published data, while not of high quality, does not support this concern in terms of breast milk quality or weight and growth of neonates [2].

LARC does not require antibiotic prophylaxis for placement, can be offered to women with a history of ectopic pregnancy, can be used in an adolescent population, and has very few contraindications. Thus, almost all women are eligible for LARC [2].

### Efficacy

According to the CDC, WHO, and ACOG, LARC is safe, but is it also effective? In a high-income population, a large study was performed in almost 7500 women comparing outcomes of participants who were given the contraceptive method of their choice at no cost, including short-acting as well as LARC methods [8]. The unintended pregnancy rate after LARC in the population was 0.27 per 100 participant-years compared to 4.55 in women using pills, patch, or ring. This resulted in a hazard ratio of 21.8 when adjusted for age, education, and history of unintended pregnancy [8]. The authors conclude that LARC is a more effective method of contraception than short-acting methods, including use in adolescent populations [8].

A review of immediate postpartum provision of LARC in high-income settings also found that these methods are safe, effective, and can reduce unmet need for contraception in the postpartum period [9]. The review concludes that IUDs inserted at the time of both vaginal and cesarean deliveries are more likely to be in situ 6–12 months postpartum than those placed at the postpartum visit 4–6 weeks after delivery [9]. They also found longer inter-pregnancy intervals in women using LARC in the immediate postpartum setting, and recommend the use of these methods regardless of impact on breastfeeding [9].

## Introduction

The background section of this paper established how LARC is defined, which contraceptives it includes, how it is recommended for use in the immediate postpartum period, and how safe and effective it is when used in real-world settings. Most data on these topics come from high-income countries (HIC). However, the focus of this review is on how LARC is currently being applied in the immediate postpartum setting in LMIC where availability of, access to, and utilization of LARC are less consistent for women in the postpartum setting.

## Methods

The objective was to review all literature published from LMIC and summarize the findings. This review, while not a systematic review, did attempt to find all published manuscripts on the use of postpartum IUDs and implants currently in production. The search included the words “postpartum”, “IUD”, “intrauterine”, “implant”, and brand names for all devices currently in production. Studies included in the review involved work in sub-Saharan Africa, Southeast Asia, and Latin America. All studies mentioned are included in the references section. Countries are grouped into income categories by the World Bank, which groups WHO member states into income categories based on gross national income per capita. For 2016, LMIC encompasses countries with gross national income per capita between $1006 and $12,235 [10].

### Demand and unmet need for modern contraception

Globally, unintended pregnancy contributes significantly to maternal morbidity and mortality, especially in LMIC settings [1]. The WHO estimates that 225 million women around the world desire modern contraception to delay or prevent future childbearing, but do not have access [11]. A review on the determinants of unmet need for family planning in LMIC defines the term as “the proportion of women wishing to limit or postpone childbirth, but not using contraception” [12]. The review found that among 26 quantitative and 8 qualitative studies, the unmet need for family planning in LMIC ranged from 20% to 58% with older age and higher educational level reducing unmet need, and a higher number of children increasing unmet need [12]. Primary reasons for non-use of modern contraception included an unsupportive partner or health concerns regarding adverse effects of the medications. These findings were not specific to LARC or the postpartum period, but addressed unmet need for modern contraception, generally [12].

**Fig. 1:**
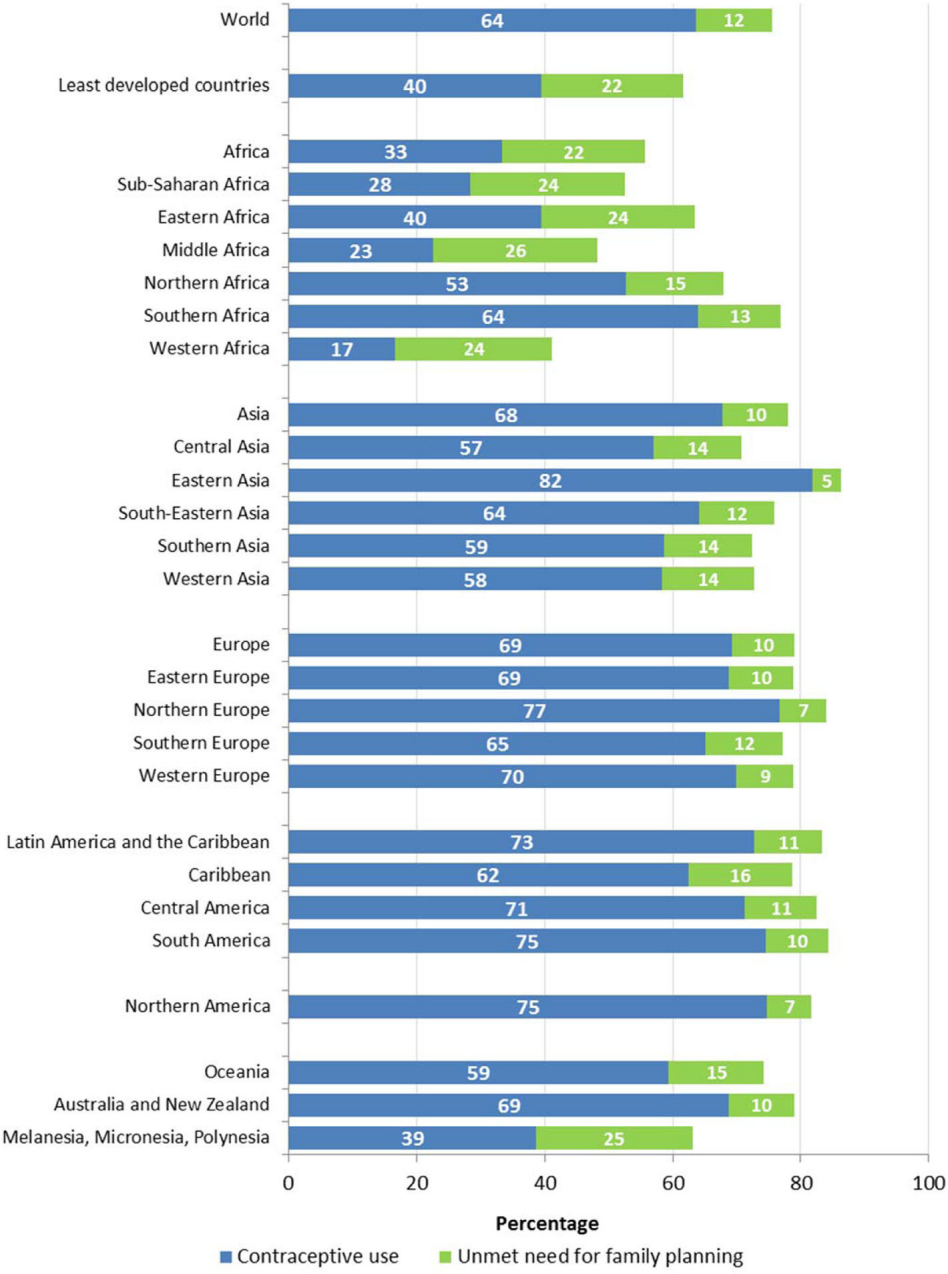
Contraceptive prevalence and unmet need for family planning among married or in-union women aged 15 to 49 years, 2015

Figure 1, reproduced with permission from the United Nations, illustrates the percentage of married or cohabitating women using contraception and with an unmet need for contraception, by region, around the world [4]. Another resource, looking at data from Demographic and Health Surveys (DHS) reported that the average level of unmet need in the 16 countries reviewed was 47% ranging from a high of 82% in Ghana to a low of 23% in Morocco. [13] The WHO identifies sub-groups at highest risk for unmet need for contraception to include adolescents, migrants, urban slum dwellers, refugees, and women in the postpartum period [14].

Of note, delivery in the facility setting appears to be increasing [15]. This finding is inferred from a recent WHO publication reports that between 1990 and 2014 the global average CS rate increased from 12.4% to 18.6% with rates ranging, depending on region, between 6 and 27.2%, and rising at an average rate of 4.4% per year [15]. Interestingly, it is not clear that this increase in facility births has translated to increased placement of postpartum contraception in facility settings, but is a potential point of entry in the healthcare system to target interventions [16].

### Reasons for unmet need for contraception in postpartum period in LMIC

The Guttmacher Institute review on unmet need for postpartum contraception in LMIC asserts that the primary reasons given for non-use in this timeframe are the protection afforded to women by lactational amenorrhea and abstinence [13]. Women, for cultural reasons, may not be sexually active in the immediate postpartum period and may experience lactational amenorrhea during breastfeeding [13]. Lactational amenorrhea ranges, on average, from 3 to 4 months to 20 months in sub-Saharan Africa (SSA) [13]. Therefore, the timeframe during which a woman may be considering postpartum contraception varies by population and individual, which can make postpartum family planning programs difficult to design and administer [13].

Another reason unmet need for contraception in the postpartum period is high in LMIC is because postpartum visits are not common, and if women did not deliver in a facility, they will have little interaction with the healthcare system to consider contraception before they become fertile again [17]. Additionally, besides requiring the woman to access the system to obtain LARC, the healthcare system needs to provide the devices. This involves appropriate counseling ante- and postnatally, a consistent supply of the devices, and providers that are trained and available to place LARC in the postpartum setting [18]. A recent study from Nigeria found that in the poorest quintile, women had a 35% unmet need for contraception [19]. This finding is largely due to the fact that LARC is not offered in many facilities that poor women frequent and stock-outs are common, which are issues seen in many LMIC [19].

Significance of LARC in LMIC: Implementing strong family planning programs in LMIC is a global health priority. This is reflected in the Millenium Development Goals, goals four and five, which focus on reducing maternal and child morbidity and mortality, incorporate increasing family planning utilization as an essential method to achieve these aims. [12] Unmet need for contraception results in adolescent, unintended, and short interval pregnancies, as well as unsafe abortion, all of which can result in increased maternal and neonatal mortality [12]. Data has shown that pregnancies conceived less than twenty months following a prior birth are at increased risk of low birth weight, preterm birth, stillbirth, and neonatal and infant mortality [13]. Additionally, a 52-country DHS survey showed that children born less than two years after a sibling have a 60% increased risk of death at less than one year of age compared to those born three to five years after another child [13]. For reasons related to adverse pregnancy and abortion outcomes, as well as the social, educational, and economic impacts of unintended or undesired pregnancy, contraception in general, and LARC specifically, have an important role to play in LMIC.

### Postpartum LARC in LMIC

#### Utilization

Currently, in LMIC, LARC use is variable. In Nairobi, Kenya’s urban slums, a study found that LARC methods were the least adopted methods during the first year postpartum, with only 4% of women opting for implants and even fewer choosing an IUD [20]. However, the study did show that these were the least discontinued methods [20]. Similarly, a study from Ethiopia that interviewed almost 900 women in the postpartum setting, found that 1.8% of those currently using contraception had opted for an IUD and 0.2% for an implant [21]. In Malawi, LARC use was higher at 14% with a statistically significant higher continuation rate at 6 months postpartum [22]. Studies from Southeast Asia (SEA) show similar utilization rates to SSA. A study from Pakistan looking at use of modern contraceptive methods in postpartum women found that out of the 27% of women who opted for a contraceptive method after delivery, 4.8% chose to use an IUD and 3.1% an implant [23]. A study of 1049 postpartum low-income Indian women found that 162 (15.4%) of them were using contraception; of these, 2.9% had an IUD in situ, and no women were using a contraceptive implant [24]. Overall, from the data reviewed, use of LARC appears to be utilized in less than 15% of postpartum women in LMIC.

#### Characteristics associated with postpartum LARC utilization

A number of studies have evaluated which characteristics make a women more or less likely to utilize postpartum contraception, with differing results. A study from Ethiopia found that in 703 participants, characteristics associated with LARC usage were resumption of menses, age < 24 years, interval of 7–9 months post-delivery, and having had antenatal care; about 11% appear to have opted for the implant and about 3% for the IUD [25]. A similar study from Uganda of almost 3300 women found that women who were more likely to utilize postpartum contraception had the following characteristics: higher than a primary level of education, highest wealth status, Protestant, aged 25–29, already had 3–5 children, had been exposed to information about family planning services via the media, were seen for post-delivery care within 1–2 days of delivery, and had their birth attended by a skilled attendant [26]. This study did not specify the rate of LARC use among the study population. Another Ugandan study found that the additional characteristics of prior use of contraceptives and partner involvement in the decision-making process were statistically significant predictors of contraceptive use since delivery, but again did not specify the type of contraceptives utilized [27]. In Pakistan, the characteristics associated with postpartum contraceptive use included women who had delivered in a facility, those who had received information about birth spacing, those living in a community with high antenatal care utilization rates, and those who lived in less impoverished areas [23]. In India, among 400 participants, characteristics that predicted non-use of postpartum contraceptives were inadequate knowledge of contraceptive options and fear of side effects [28]. Understanding the specific predictors of use and non-use of postpartum contraceptives, and specifically LARC, in some LMIC settings may help identify targets for interventions to increase uptake in these countries, more generally.

#### Efficacy

Understanding the safety and efficacy of LARC use in LMIC is essential before programs to increase utilization are implemented. A study from India on postpartum copper IUD placement (after both vaginal and cesarean delivery) found that none of the 434 women who continued to use the IUD were pregnant by 6–18 months postpartum [29]. Continuation rates of the device were 81% by the end of the study [29]. A study from Kenya studied continuation rates of the implant, but did not comment on efficacy in terms of pregnancy rates during the follow-up period; the authors found that of the 97 women who were followed for a year, 79% had continued use of the implant up to that point. [30] A review from the United States Agency for International Development looks at Demographic and Health Surveys and focused on uptake and discontinuation of LARC in LMIC [31]. This document reports that, “On average, within the first year of use, 9 percent of women discontinue using implants, 15 percent discontinue IUDs, and 32 percent discontinue injectables,” although this information is not specific to LARC placed in the postpartum period [31].

#### Side effects

The study previously mentioned from India on postpartum copper IUD placement found that in 434 women who received the device, 190 experienced a complication (bleeding 23.5%, expulsion 9%, inability to visualize strings 11.3%) [28]. A recently published study from Uganda where authors conducted focus groups on factors that influenced respondents use of LARC found that women who chose short-acting over long-acting methods cited side effects of LARC as their primary reason for doing so, although this was not specific to the postpartum setting [32]. Side effects mentioned were excessive bleeding and lack of periods [32]. In a study of over 1200 Pakistani women who chose LARC methods for contraception, also not specifically in the postpartum setting, found that about 25% of participants complained of side effects related to their device, citing bleeding and pain most commonly [33]. About 30% of patients sought follow-up visits for side effect-related complaints during the twelve-month follow-up period [33]. How side effects from LARC use affect patients in the postpartum setting, specifically, deserves further attention.

#### Satisfaction

As important to increasing effective family planning programs as are utilization and continuation of postpartum contraceptives, patient satisfaction with the method chosen may be more important. Since many contraceptives have unwanted side effects, finding the right method for a particular women is essential. A study of over 2700 Indian women who opted for a postpartum copper IUD after facility delivery were assessed for their satisfaction immediately status-post placement of the device and six weeks later at their follow-up visit [34]. The mean age of participants was 24 years old, >50% had one living child, and one quarter of the patients had no formal education [34]. 99.6% of the women were satisfied with the IUD at the time of insertion and 92% were satisfied at the six week follow-up visit, at which point the rate of expulsion was 3.6% [34]. A study from Kenya looked specifically at patient’s satisfaction with postpartum LARC in the year following device placement, including the implant and both types of IUD [35]. The study was a longitudinal cohort study of 313 women (205 used the subdermal implant, 93 used the LNG IUD, 15 opted for the copper IUD) whose LARC was placed in an interval fashion (6–12 weeks after delivery) and not immediately postpartum within 48 h [35]. The authors declined to publish results for the copper IUD users because they felt the sample size was too small; for the LNG IUD and implant, they found that continuation rates were 89.1% and 91.8%, respectively [35]. 87% of patients reported being ‘very satisfied’ with the LNG IUD and 75% with the implant at 6 months postpartum; 83.9% and 87.3% were ‘very satisfied’ after a year, respectively [35]. The authors felt the LNG IUD compared favorably to the implant both in terms of satisfaction and continued use [35]. These results suggest that LARC performs well in LMIC in terms of the patient experience.

#### Impact on pregnancy outcomes

Studies from HIC have shown that postpartum LARC can positively impact pregnancy outcomes. For example, a prospective observational study of patients in Colorado found that with immediate postpartum implant insertion, there was a 2.6% pregnancy rate one year post-placement in the intervention group compared to an 18.6% pregnancy rate among the control group [36]. Downstream benefits of using LARC in this population also appeared to include a 2% decrease in preterm birth, which was an unexpected but highly beneficial outcome [36]. This suggests that effective postpartum LARC programs may have an impact on adverse pregnancy outcomes such as preterm birth. While studies from HIC are not the focus of this part of the review, we think it is helpful to summarize the research that has been done in this area to guide future work in LMIC, as studies in this area do not appear to have yet been performed.

#### Barriers to uptake

We have shown that utilization of postpartum LARC in LMIC overall is poor at rates less than 15%. However, according to the few studies that exist, LARC efficacy is good and satisfaction is high, and there is data from HIC that the impact of the contraceptives on pregnancy outcomes can be beneficial. The next question becomes, what are the barriers to postpartum LARC uptake in LMIC? We have noted that stock-outs, alternative methods of contraception (lactational amenorrhea and abstinence), and poor postpartum healthcare may contribute to poor utilization of postpartum LARC in LMIC. A study from rural Ghana attempted to clarify barriers to IUD uptake. They interviewed pregnant, postpartum, and reproductive-aged women, as well as males and healthcare workers [37]. While not specific to postpartum LARC use, the study found that lack of IUD-specific knowledge, provider discomfort with placement of the device, and poor contraceptive counseling were reasons IUD uptake was poor [37]. The authors recommended that interventions to educate and train providers, increased peer modeling, and improve stocking of IUDs in the community could increase uptake [37]. Incentives to place IUDs, which involve more effort than say an injectable, may also be required.

A study from Rwanda published results of in-depth interviews with women and their partners about barriers to modern contraceptive use postpartum [38]. They concluded that fertility and partner-related factors were associated with non-use of modern contraceptives, especially in the postpartum period because patients had a poor understanding of their fertility during that time [38]. As a result, the authors recommended increasing educational campaigns aimed at couples and increasing information about postpartum contraceptive needs [38]. This is another area that could benefit from more study, especially as it relates specifically to postpartum LARC use in LMIC.

#### Interventions to increase utilization

This segment of the review focuses on what has been done to improve use of postpartum LARC in LMIC. A number of studies have tried different techniques in both SSA and SEA (Southeast Asia). A review on interventions to improve postpartum family planning use in LMIC found 35 articles focused on interventions to improve postpartum contraception [39]. The authors found eight studies of antenatal interventions and while they felt the data was not of sufficient quality to draw a conclusion, they did suggest “high-intensity” antenatal counseling might affect postpartum contraceptive uptake [39]. All of these studies focused on some type of antenatal counseling program and were conducted in SSA, SEA, and the Middle East.

Eleven studies of postpartum interventions were evaluated, and while again the authors questioned the quality of the evidence, they assert that a single 20-min postnatal counseling session may be able to increase contraceptive uptake [39]. These interventions also focused on postpartum counseling and were conducted all over the world including Latin America, SSA, and Asia.

Combined ante- and postnatal interventions were also reviewed in ten studies. While the authors found that they all showed a substantial impact on contraceptive uptake sometime in the first year postpartum, they questioned whether it was possible to determine which component(s) of the interventions was effective and whether resources were being wasted on combined interventions [39]. These combined interventions were more involved and included not only counseling but home visits, partner counseling, and community mobilization [39]. Studies were included from all major world regions.

The final intervention type they reviewed was the integration of family planning services with other healthcare services, such as childhood immunization programs. [39] They felt that while these interventions had the most potential to be successful, results were not impressive and data were lacking on the effectiveness of these programs. [39] The authors conclude that the “ideal strategy for improving postpartum family planning is to incorporate contraceptive advice and services across the continuum of reproductive healthcare,” and that no single intervention or time point was maximally effective at improving postpartum contraceptive uptake [39]. While this review did focus on the postpartum setting, it reviewed all contraceptive methods and was not specific to LARC.

Recently, a review was published on the promotion of intrauterine contraception in LMIC [16]. The authors reviewed and interpreted interventions studied to promote uptake of IUDs in LMIC; based on their narrative review of the literature they concluded that the evidence-base is weak and that no clear strategy exists for increasing utilization in LMIC [16]. They suggest this is due to adverse perceptions of IUDs by providers and patients and that most interventions are not funded by LMIC governments, but rather international organizations [16].

#### Economic and cost implications for LARC programs in LMIC

The final part of this review is to address the cost and economic implications of scaling up LARC programs in LMIC. Unfortunately, sophisticated cost-effectiveness analyses have not been published from LMIC settings, suggesting this is another area ripe for research. However, NICE has published a clinical practice guideline on the cost-effectiveness of LARC use in the United Kingdom [40]. LARC, in this review, not only included both IUDs and the implant, but the depot medroxyprogesterone acetate injection, as well. The study found that LARC methods were the most effective and least costly, with depot medroxyprogesterone acetate and the LNG IUD being the least cost-effective of the methods studied due to discontinuation, which was a key determinant of their cost-effectiveness [40]. Of note, the incremental cost-effectiveness of the implant, which is considered the most effective LARC method per NICE, as compared to the IUD, which is the cheapest, was £13,206 per unintended pregnancy averted for one year of use [40]. The authors conclude that interventions to improve patient satisfaction and continuation of LARC methods will improve cost-effectiveness even further [40].

## Conclusions

The main conclusion of this review is that immediate postpartum LARC has been proven to be a safe and effective means of family planning not only to improve individual health, social, and economic outcomes, but also to achieve global priorities for public health. However, the review does highlight the fact that in each of the different topic areas addressed, there is a lack of high-quality data from LMIC, and that more research needs to be done on making the devices available, promoting programs for large-scale implementation, monitoring and evaluating the impact of LARC on fertility and subsequent pregnancy outcomes, and the cost and cost-effectiveness of such device use in LMIC settings. Additionally, the literature could benefit from more research on the limitations of the use of LARC in LMIC. It may be that these methods are inappropriate for use in such settings given lack of provider availability or training, potential for uterine perforation with placement of IUDs without the opportunity for adequate follow-up, and side effects may be either more pronounced or unacceptable in certain LMIC patient populations. The proof of concept groundwork has been laid that postpartum LARC is acceptable for use in LMIC. Now it is important to invest in the utilization of these products and study if they have the highly beneficial effects on individual and group health, social, and economic outcomes, expected.

## Abbreviations

ACOG: American College of Obstetrics and Gynecologists
CDC: Center for Disease Control and Prevention
DHS: Demographic and Health Surveys
FDA: Food and Drug Administration
HIC: High-income countries
IUDs: Intrauterine devices
LARC: Long-acting reversible contraception
LMIC: Low- and middle-income countries
LNG: Levonorgestrel
NICE: National Institute for Healthcare Excellence
SEA: Southeast Asia
SSA: Sub-Saharan Africa
UN: United Nations
USA: United States of America
WHO: World Health Organization
